# Conserved DNA sequence analysis reveals the phylogeography and evolutionary events of *Akebia trifoliata* in the region across the eastern edge of the Tibetan Plateau and subtropical China

**DOI:** 10.1186/s12862-024-02243-0

**Published:** 2024-04-23

**Authors:** Qing Dong, Yongle Zhang, Shengfu Zhong, Qiuyi Zhang, Hao Yang, Huai Yang, Xiaoxiao Yi, Feiquan Tan, Chen Chen, Peigao Luo

**Affiliations:** Key Laboratory of Plant Genetics and Breeding at Sichuan Agricultural University of Sichuan Province, Sichuan Agricultural University, Chengdu, China

**Keywords:** *Akebia Trifoliata*, Intraspecific evolution, ITS sequence, Migration, Phylogeography, *rps16*

## Abstract

**Background:**

The eastern edge of the Qinghai‒Tibet Plateau (QTP) and subtropical China have various regions where plant species originate and thrive, but these regions have been the focus of very few integrative studies. Here, we elucidated the phylogeographic structure of a continuous and widespread *Akebia trifoliata* population across these two regions.

**Results:**

Sixty-one populations consisting of 391 genotypes were examined to assess population diversity and structure via network distribution analysis, maximum likelihood phylogenetic tree reconstruction, divergence time estimation, demographic history inference, and ancestral area reconstruction of both conserved internal transcribed spacer (ITS) and chloroplast (*rps16*) DNA sequences. The results showed that the ITS region was more variable than the *rps16* region and could be suitable for studying intraspecific phylogeography. The *A. trifoliata* population displayed high genetic diversity, genetic differentiation and obvious phylogeographical structure, possibly originating on the eastern QTP, expanding during the last glacial-interglacial cycle, diverging in the early Pleistocene and middle Pleistocene, and extensively migrating thereafter. The migration route from west to east along rivers could be largely responsible for the long-distance dispersal of this species, while three main refuges (Qinba Mountains, Nanling Mountains and Yunnan-Guizhou Plateau) with multiple ice shelters facilitated its wide distribution.

**Conclusions:**

Our results suggested that the from west to east long migration accompanying with the minor short reciprocal migration in the south-north direction, and the three main refuges (the Qinba Mountains, Nanling Mountains and Yunnan-Guizhou Plateau) contributed to the extant geographical distribution of *A. trifoliata*. In addition, this finding also strongly reduced the discrepancy between glacial contraction and postglacial expansion and the in situ survival hypothesis by simultaneously considering the existence of many similar climate-related ecological niches and migration influences.

**Supplementary Information:**

The online version contains supplementary material available at 10.1186/s12862-024-02243-0.

## Background

China’s present-day topography is well known for being elevated in the west and low in the east, which could have resulted from the rapid uplift of the Tibetan Plateau (TP) during the Quaternary Period [1]. As a main driving force of eco-environmental evolution, rapid changes have led to corresponding alternations in both geomorphic and climate types and consequently generated various sources and reservoirs of biological diversity in the region surrounding the TP [2]. Further analysis supported that the climate oscillations accompanying this process resulted in alternation between contraction and expansion due to cyclical variations in glacial intervals, which had an important influence on the current distributions of extant species [3]. Recently, many glacial refugia found in mainland China during the species conservation process also well supported this view [4]. For sedentary and/or special species, populations close to glacier refugia generally have greater genetic diversity than those far from them [5]. Therefore, scientists can infer the evolutionary history of a given species according to the distribution of its genetic information.

In the region of the eastern edge of the TP, the alternating distribution of a series of spectacular north‒south trending mountains and rapidly rushing rivers results in various deep valleys, in which numerous species occur due to the large variation in altitudes. Regions harbouring both ancient and newly originated species are the most famous biodiversity cradles in the world [6]. Studies have suggested that many species retreated here for shelter during the ice ages but migrated to other places after the ice ages since the beginning of the Quaternary [7–9], which indicated that the area was a major shelter and possibly an important centre of species origin. In addition, the migration of species, especially vascular plants, directly across the TP would have been impeded by various high mountains with perennial frozen earth [10], indicating that subtropical China, both south of the Qinling Mountains and Huaihe River Line and east of the TP [11], is also an ideal geographical area for investigating species’ refuge locations and migration routes.

Species in these areas are useful for biologists studying evolutionary events [12], and there have been various reports on the evolution of species in the region. Based on the evidence from both ancient vegetation and systematic geography, two main models have been proposed to describe the refugia and postglacial population dynamics of plant species in subtropical China [13]. One is the glacial contraction and postglacial expansion model (CE model) [14], which asserts that species experienced sharp contraction during glacial periods and quick expansion after glacial periods. For example, reconstruction of the palaeontological community showed that the subtropical evergreen broad-leaved forest in China retreated c. 1000 km to the south during the last glacial maximum (LGM, c. 0.021 − 0.018 Ma), while large-scale expansion occurred in the northern high-latitude area after that period [14, 15], which provided direct evidence supporting the CE model. In addition, comparative analysis revealed that southern populations usually had greater genetic diversity than northern populations, possibly resulting from the greater accumulation of ancient and private alleles during the longer evolutionary history compared with that of the northern population [16, 17]. The other is the in situ survival model (ISS model), which considers that there are many shelters during glacial periods and relatively few local expansion events after glacial periods [18–20]. Most of the evidence supporting this model mainly comes from research on deciduous broad-leaved forest plants [6, 13, 21–23]. In fact, the CE model ignores the possibility of many ecological niches with similar climate conditions, while the ISS model poorly accounts for migration effects.

In addition, different plant types can have different evolutionary histories. Thus, further studies of population evolutionary histories across the eastern edge of the TP and in subtropical China with new plant types would be valuable for understanding species history. However, few studies have simultaneously considered populations across the two regions [24, 25]. Broad-leaved forest trees have received little attention because there are very few with wide and continuous distributions in the two regions due to large variations in climate, elevation, and landforms [26], while other plant types, such as vines, have not received much attention in phylogeographic research due to their weakened role in forests compared with that of tall, large woody trees. Furthermore, the two regions together account for one-third of China’s land area [27], so it is difficult to simultaneously sample at this large scale due to a shortage of available ex situ conservation genetic resources. In addition, underdeveloped road networks on the eastern edge of the TP due to natural barriers are also an important reason for the few samples collected in the past. With the establishment of living germplasm banks of various woody perennial plants and the improvement of transportation infrastructure, systemically studying evolutionary history by integrating samples from the two regions is becoming feasible.

*Akebia trifoliata* (three-leaf *Akebia*) is a woody perennial climbing vine in the family Lardizabalaceae [28]. Various studies have suggested that *A. trifoliata* has many advantages for elucidating the phylogeography of mainland China. First, according to the records from the Plants for a Future database (www.pfaf.org), it originated in China (https://pfaf.org/user/Plant.aspx? LatinName = Akebia + trifoliata), although South Korea and Japan also have populations of *A. trifoliata* [29], and it is continuously distributed from the eastern edge of the TP to the eastern coast of mainland China; therefore, *A. trifoliata* is a typical species of the region spanning the eastern edge of the TP and subtropical China [28]. In addition, characteristics such as a small genome [30], a relatively short juvenile stage, a large breeding coefficient, cross-pollination and numerous discernible phenotypic traits make *A. trifoliata* an ideal model plant, especially for perennial woody species [31, 32]. Third, the availability of sufficient wild germplasm from the whole mainland of China [33] is highly advantageous for systemically studying the phylogeography of *A. trifoliata*. There have been few reports on the genetic diversity of *A. trifoliata*, but the employed samples were narrow in scope and usually small in size [34]. Therefore, further study of phylogenetic relationships will be valuable for understanding the evolutionary history of *A. trifoliata*. In the present study, conserved DNA sequences, including those of the maternally inherited chloroplast gene *rps16* and the biparentally inherited nuclear ribosomal internal transcribed spacer (ITS), were used to explore the genetic diversity and population structure of *A. trifoliata* in the region across the eastern edge of the TP and subtropical China. Our objectives were to determine the locations of the main shelters of *A. trifoliata*, to infer the migration route, and to identify the evolutionary model responsible for the distribution of extant *A. trifoliata* populations.

## Methods

### Plant genotypes

A total of 391 genotypes of 61 populations from the *A. trifoliata* ex situ conservation germplasm bank located at the Chongzhou Research Station of Sichuan Agricultural University [31] were chosen for the study. These genotypes originated from a large geographical area spanning from 99°57′E to 120°11′E and from 24°49′N to 34°42′N in mainland China.

### DNA extraction, amplification and sequencing

Total genomic DNA was extracted according to the previously published CTAB method. The *rps16* chloroplast gene and ITS were selected as maternally and biparentally inherited molecular markers, respectively [35, 36]. The corresponding primers were synthesized and then subjected to polymerase chain reaction (PCR) according to a previously described reaction system and program [31]. Sequencing reactions were conducted with corresponding forward and reverse primers commercially provided by Tsingke Biotechnology Co., Ltd. (Chengdu, China).

### Population genetic diversity and genetic differentiation

For the *rps16* and ITS data, the sequence was first manually edited using BioEdit 7.0.1 [37] and then aligned with MAFFT 7.2.2 software [38]. Finally, the conserved region of the aligned sequence was extracted using Gblock v 0.91b [39]. We used DnaSP 5.10 software to calculate genetic information statistics, including haplotype number, Hd and π, of the conserved sequence [40]. In addition, we used PERMUT 2.0 [41] to calculate the h_T_ and h_S_ between populations with 1,000 repeats and to assess the difference between the G_ST_ and N_ST_ of distinct populations, excluding populations with sample sizes less than 3. Analysis of molecular variance (AMOVA) and correlation analysis between genetic distance and geographical distance were performed by Arlequin 3.0 [42] and by GenAlEx 6.5 [43], respectively.

### Phylogenetic analyses and molecular dating

The haplotype (ITS haplotypes and *rps16* haplotypes) network diagram was constructed using PopART 1.7 [44], and a sampling distribution map was constructed using ArcGIS 10.2 [45]. ML trees were reconstructed according to conserved haplotype sequences using PhyloSuite 1.2.2 [46] with the best-fit models (TPM3 + F + I and TIM3 + F + R2 for *rps16* and ITS, respectively) produced from the AIC program of ModelFinder software [47], in which *Archakebia apetala* was used as the outgroup.

The divergence time of the haplotype lineages of *A. trifoliata* was determined with a secondary calibration method by BEAST 2 [48]. The first step was to determine the crown age of *A. trifoliata*. Four *matK* gene sequences of *A. trifoliata* and 15 *matK* gene sequences of 15 different species were employed by searching GenBank (Table S2), consisting of six from Lardizabalaceae, five from Menispermaceae, two from Berberidaceae, one from Eupteleaceae and one from Ranunculaceae. In addition, four calibrated time points, *Ranzania*-*Mahonia*-*Berberis* (45.0 Ma) [49], *Chasmanthera*-*Odontocarya* (33.9 Ma) [50], *Tinospora*-*Chasmanthera*-*Odontocarya* (55.2 Ma) [51, 52], and *Stephania*-*Menispermum*-*Chasmanthera*-*Tinospora*-*Odontocarya* (89.3 Ma) [53], were used. The chronogram of Ranunculales was produced using BEAST2 with the Yule model, an uncorrelated lognormal relaxed clock [54] and the TVM + F + G4 best-fit model selected by the BIC approach in ModelFinder software [47]. For each BEAST2 analysis, Markov chain Monte Carlo (MCMC) was run for 1 × 10^8^ generations, sampling once every 1000 generations, and the first 10% of the samples were removed as burn-in. The sufficiency and convergence of the sampling results were checked by Tracer 1.7.2.

In the second step, chronograms of *A. trifoliata* haplotypes were further derived using a method similar to that used to date intraspecific nodes, in which the root nodes were corrected by using the above estimated crown group age (median value) of *A. trifoliata* in the first step, and *A. apetala* was still an outgroup. GTR + F + G4 and F81 + F + G4 were the best-fit models for the ITS and *rps16* haplotypes, respectively. *A. apetala* was still an outgroup.

### Demographic history

The values of Tajima’s [55], Fu and Li’s [56] and Fu and Li’s [57] of all sequences were calculated by DnaSP 5.10 software [40], and the observed paired difference pattern (mismatch distribution) was also analysed using the same software with a constant population size model. Both the sum of squared deviations (SSD) and Harpending’s raggedness index (HRag) were analysed by Arlequin 3.0, and we further analysed population expansion only when the corresponding *p* value was greater than the 0.05 level. The expansion time was calculated using the formula T = τ/2µkg, where µ, k and g refer to the replacement rate (s s^− 1^ yr^− 1^) of each site in the ITS every year, the length of the sequence and the age of the first reproduction, respectively. In this study, a mean value of µ for shrubs and herbal plants ranging from 3.46 to 8.69 × 10^− 9^ (s s^− 1^ yr^− 1^) [58] was used as the µ value of the *A. trifoliata* ITS sequence. The g value of *A. trifoliata* is usually three years.

### Ancestral area reconstructions

Based on the chronograms of the ITS and *rps16* haplotypes, the geographical diversification of *A. trifoliata* was further reconstructed via Bayesian binary MCMC (BBM) analysis in RASP 4.x [59]. During this process, the distribution of *A. trifoliata* was divided into four regions according to natural geographical boundaries: A (the eastern Tibetan Plateau), B (central northern China), C (central China) and D (eastern China). MCMC trees were constructed with 5000 randomly chosen trees from all post-burn-in trees from BEAST2 analysis. The parameters of the BBM analyses were set as follows: null for the root distribution, 10 chains for optimization with the fixed JC model for 5 × 10^5^ cycles, the posterior distribution sampled every 100 generations, and four regions for the permitted maximum value.

## Results

### Sequence variation, genetic diversity, and genetic structure

The lengths of the *rps16* and ITS sequences obtained from 391 samples of *A. trifoliata* were 744 bp with a 33.9% G + C content and 695 bp with a 63% G + C content, respectively. We first identified a total of 22 *rps16* haplotypes (C1 ∼ C22) and 75 ITS haplotypes (R1 ∼ R75) according to 59 and 88 polymorphic sites in the *rps16* and ITS sequences, respectively. ITS sequences had greater haplotype diversity (Hd) (0.86), nucleotide diversity (π) (3.07 × 10^− 3^), total genetic diversity (h_T_) (0.88), average genetic diversity (h_S_) (0.31) and fixation index (F_ST_) (0.64) values among the 61 small populations than did *rps16* sequences (Table S1). In addition, we also found that for the ITS dataset, the G_ST_ (0.44) was lower than the N_ST_ (0.68), while for the *rps16* dataset, the G_ST_ (0.44) was larger than the N_ST_ (0.31), which indicated that the ITS dataset had an obvious phylogeographic structure, while the *rps16* dataset did not.

**Fig. 1:**
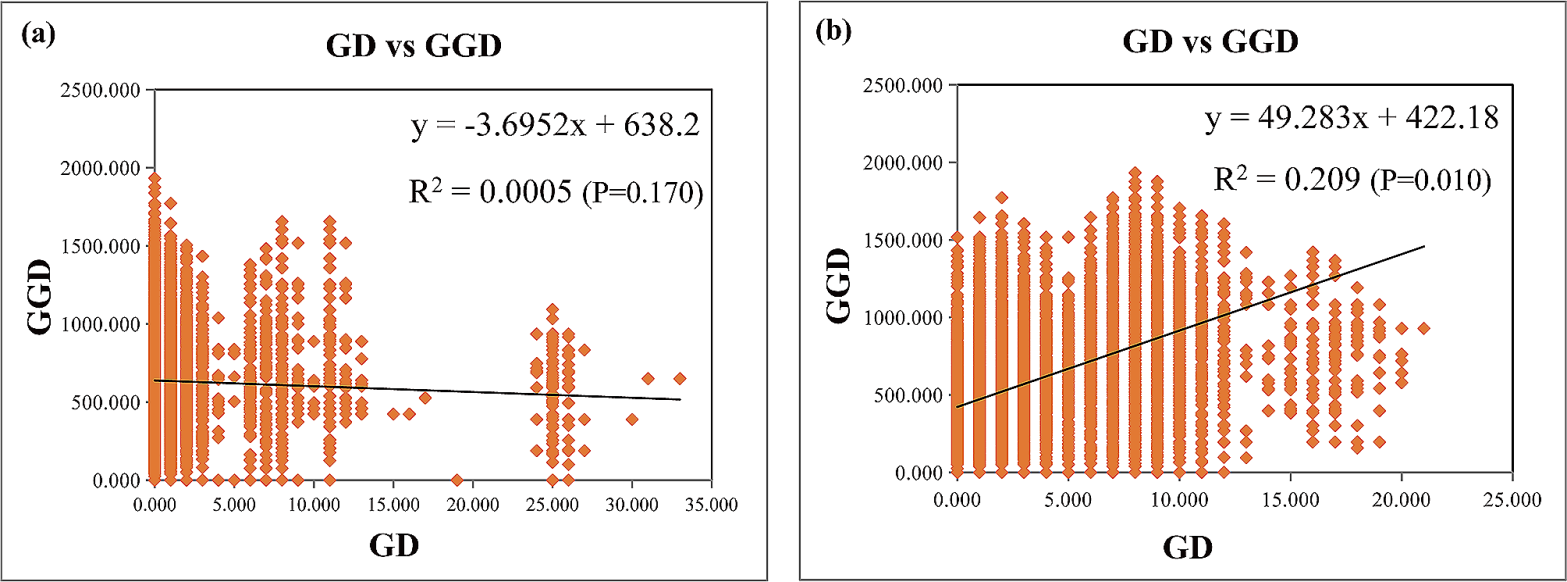
Mantel test results for the geographic distance matrix (GGD) and genetic distance matrix (GD) based on (**a**) the *rps16* dataset and (**b**) ITS dataset

In fact, the results of the Mantel test suggested that for the *rps16* dataset, there was no obvious correlation between genetic distance and geographical distance among populations (R^2^ = 0.0005, *p* = 0.17) (Fig. 1a), while for the ITS dataset, the relationship was significant at the *p* = 0.05 level, although R^2^ was only 0.21 (Fig. 1b). Furthermore, the AMOVA results showed that the variation in the ITS region mainly occurred among populations, while that in the *rps16* region mainly occurred within populations (Table 1).

**Table 1:** Molecular variance (AMOVA) of *rps16* haplotypes and ITS haplotypes for *Akebia trifoliata* populations

Source of variation		*rps16*			ITS		
	df	Ss	Vc	Pv (%)	Ss	Vc	Pv (%)
Among populations	67	30.19	0.03	7.74	593.61	1.41	64.07
Within populations	324	99.83	0.31	92.26	256.97	0.79	35.93
Total	391	130.01	0.335		850.58	2.21	
Fixation index		F_ST_ = 0.09 (*p* < 0.05)			F_ST_ = 0.64 (*p* < 0.05)		

df, degrees of freedom; Ss, sum of squares; Vc, variance components; Pv (%), percentage of variation (%)

### Network and distribution of haplotypes

**Fig. 2:**
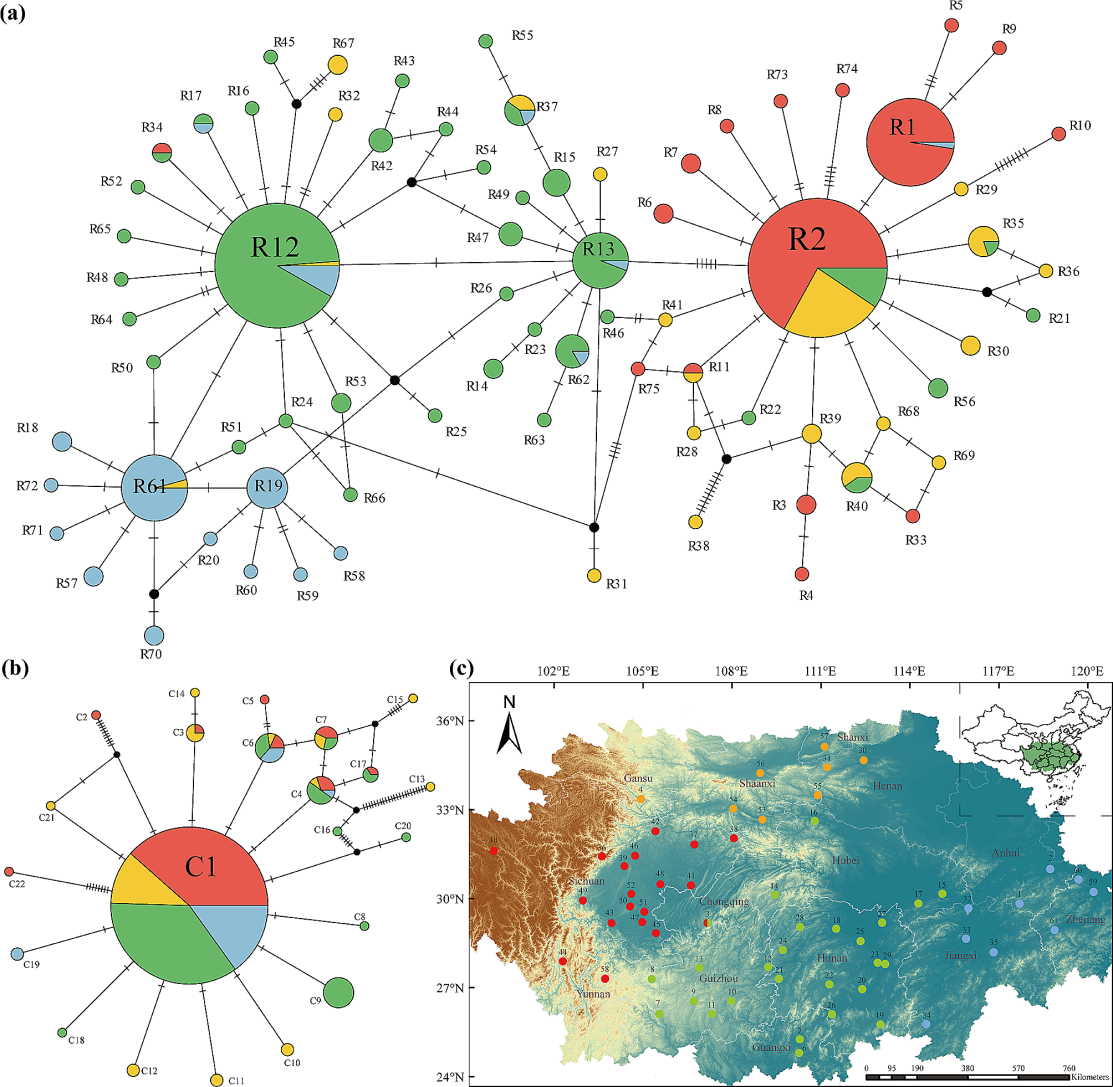
(**a**) TCS-derived network of genealogical relationships between the 75 ITS haplotypes. (**b**) TCS-derived network of genealogical relationships between the 22 haplotypes (*rps1*6). The circle size of the two network diagrams is proportional to the sample size of each haplotype. Black filled dots indicate missing haplotypes. The haplotypes from the four regions are represented by four different colours. The dashes on the straight line represent the number of abrupt steps. (**c**) Sampling distribution map of 61 populations of *Akebia trifoliata*. Red, yellow, green and blue represent the different geographical locations of A (the eastern Tibetan Plateau), B (central northern China), C (central China) and D (eastern China), respectively

The TCS network diagrams of the ITS haplotypes exhibited a multiple star-shaped radial pattern, and the haplotypes clearly corresponded to distinct geographical regions (Fig. 2a). In contrast, the distribution of *rps16* haplotypes showed a single star-shaped radial pattern (Fig. 2b), which indicated that there was no geographical structure. According to the geographical distribution of the ITS haplotypes (Fig. 2a), no haplotype was common to all regions, while 63 (84%) of the haplotypes were region specific. Among the 63 region-specific haplotypes, 12 (R3 ∼ R10, R33, R73 ∼ R75), 13 (R27 ∼ R32, R36, R38, R39, R41, R67 ∼ R69), 28 (R14 ∼ R16, R21 ∼ R26, R42 ∼ R56, R63 ∼ R66) and 10 (R18 ∼ R20, R57 ∼ R60, R70 ∼ R72) haplotypes were specific to the A, B, C and D regions, respectively (Fig. 2c and Table S1). We further found that 26 haplotypes, including 12 of the A region-specific haplotypes, 13 of the B region-specific haplotypes and one (R11) haplotype common to the A, B and C regions, were derived from R2 by one to ten steps of mutation; 28 of the C region-specific haplotypes were derived from R12 by one to four steps of mutation; and 10 of the D region-specific haplotypes were derived from R61 by one to three steps of mutation. Obviously, R2 is the ancestral haplotype because of its large proportion and wide distribution.

Moreover, we detected 22 *rps16* haplotypes, 16 (72.7%) of which were region specific, while three (13.6%) *rps16* haplotypes (C1, C4 and C6) were common to all four lineages. We also found that C1 produced four B region-specific *rps16* haplotypes (C10-C12 and C21), two C region-specific *rps16* haplotypes (C8 and C9) and one D region-specific *rps16* haplotype (C19) via one mutation step and directly produced two A region-specific *rps16* haplotypes (C2 and C22) via multiple steps of mutation. Moreover, all *rps16* haplotypes of the D region were simply derived from C1 by only one mutation step. The results suggested that C1 was the ancestral *rps16* haplotype. Overall, the distributions of both the ITS and *rps16* haplotypes revealed obvious ITS sequence divergence, while the *rps16* sequence lacked such divergence across geographical regions.

### *ML phylogenetic tree of* A. trifoliata *haplotypes*

**Fig. 3:**
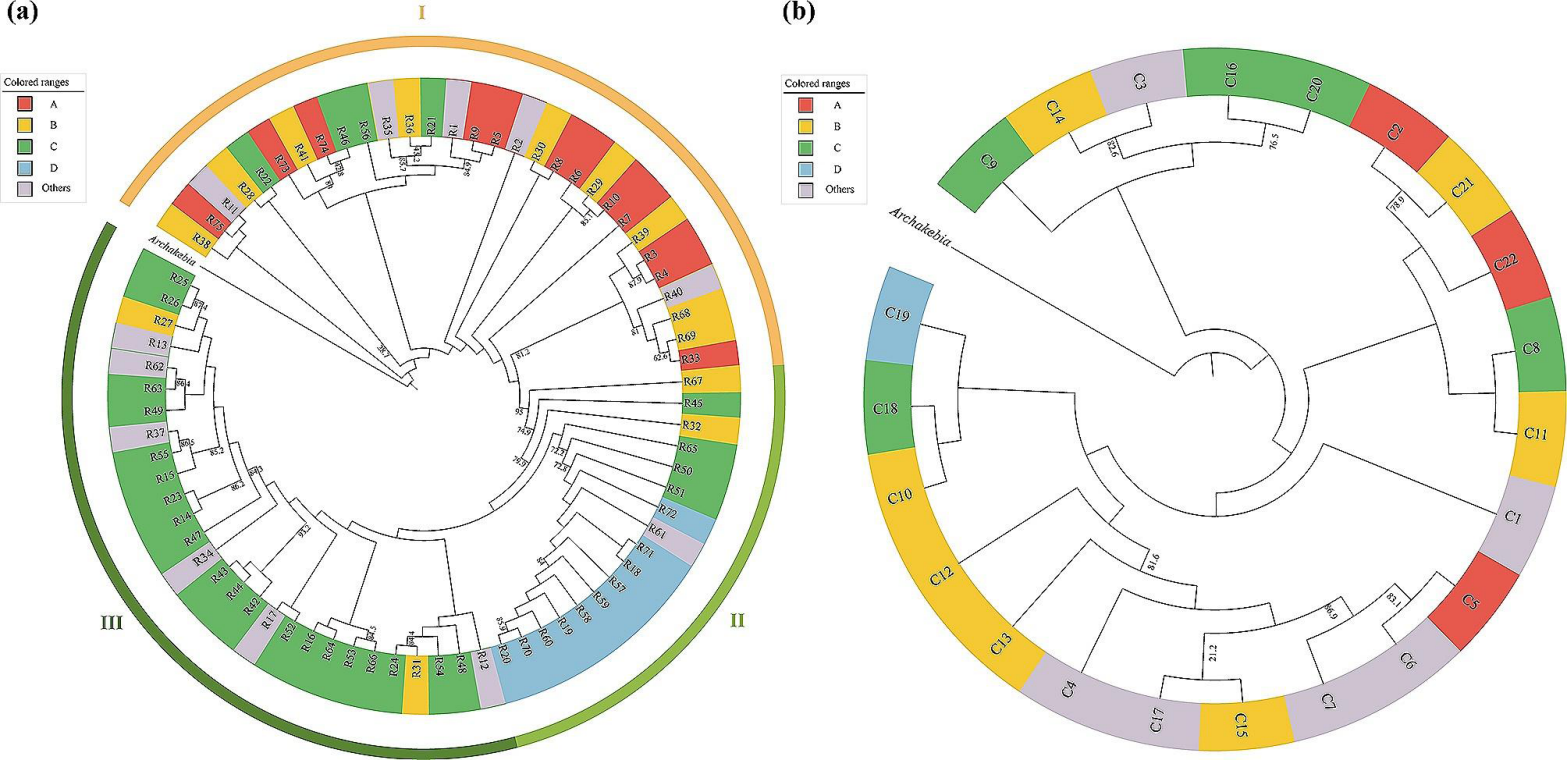
Maximum likelihood tree for *A. trifoliata* based on (**a**) ITS haplotypes and (**b**)*rps16* haplotypes; *Archakebia apetala* was the outgroup. Bootstrap probabilities are indicated on the branches. Red, yellow, green and blue in colour represent A (the eastern Tibetan Plateau), B (central northern China), C (central China) and D (eastern China) region-specific haplotypes, while others in grey represent haplotypes from two to four different regions. I, II and III are the three major branches of the ITS haplotype ML tree (**a**)

The maximum likelihood (ML) phylogenetic tree of all 75 *A. trifoliata* ITS haplotypes with *A. apetala* as an outgroup had three major branches: I, II and III (Fig. 3a). Branch I mainly consisted of all (12) specific haplotypes from the A region, most (nine out of 13) specific haplotypes from the B region, four specific haplotypes (R21, R22, R46 and R56) from the C region and five other common haplotypes (R1, R2, R11, R35 and R40) in two or three regions; branch II mainly consisted of all (10) specific haplotypes from the D region, two specific haplotypes (R32 and R67) from the B region, four specific haplotypes (R45, R65, R50 and R51) from the C region and one common haplotype (R61) in the B and D regions; and branch III mainly consisted of almost all (24 out of 28) specific haplotypes from the C region, two B region-specific haplotypes (R27 and R31) and six other common haplotypes (R12, R13, R17, R34, R37 and R62) in two or three regions. In contrast, there was no obvious branch on the ML phylogenetic tree of all 22 *A. trifoliata rps16* haplotypes (Fig. 3b), in which the haplotypes were evenly distributed among the four regions.

### *Divergence time of* A. trifoliata *haplotypes*

**Fig. 4:**
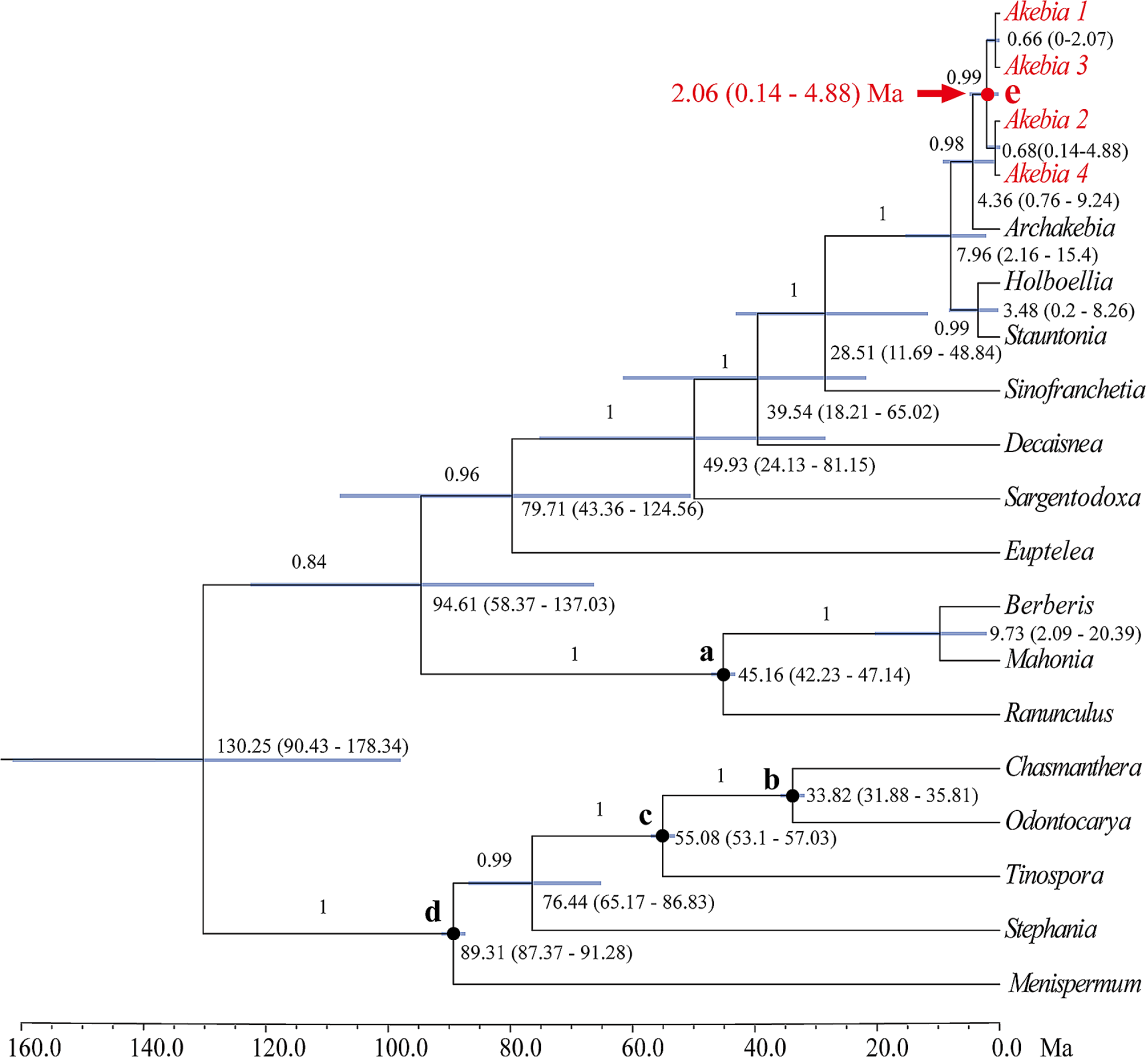
BEAST2-derived chronogram of Ranunculales based on *matK* sequences with calibration points denoted by nodes a-d. The blue bars indicate the 95% HPD credibility intervals for node ages (in Myr ago, Ma). The age estimate (Ma) (mean 95% highest posterior density: HPD) for each node is shown beside the nodes. The posterior probability values are labelled on each branch in turn

**Fig. 5:**
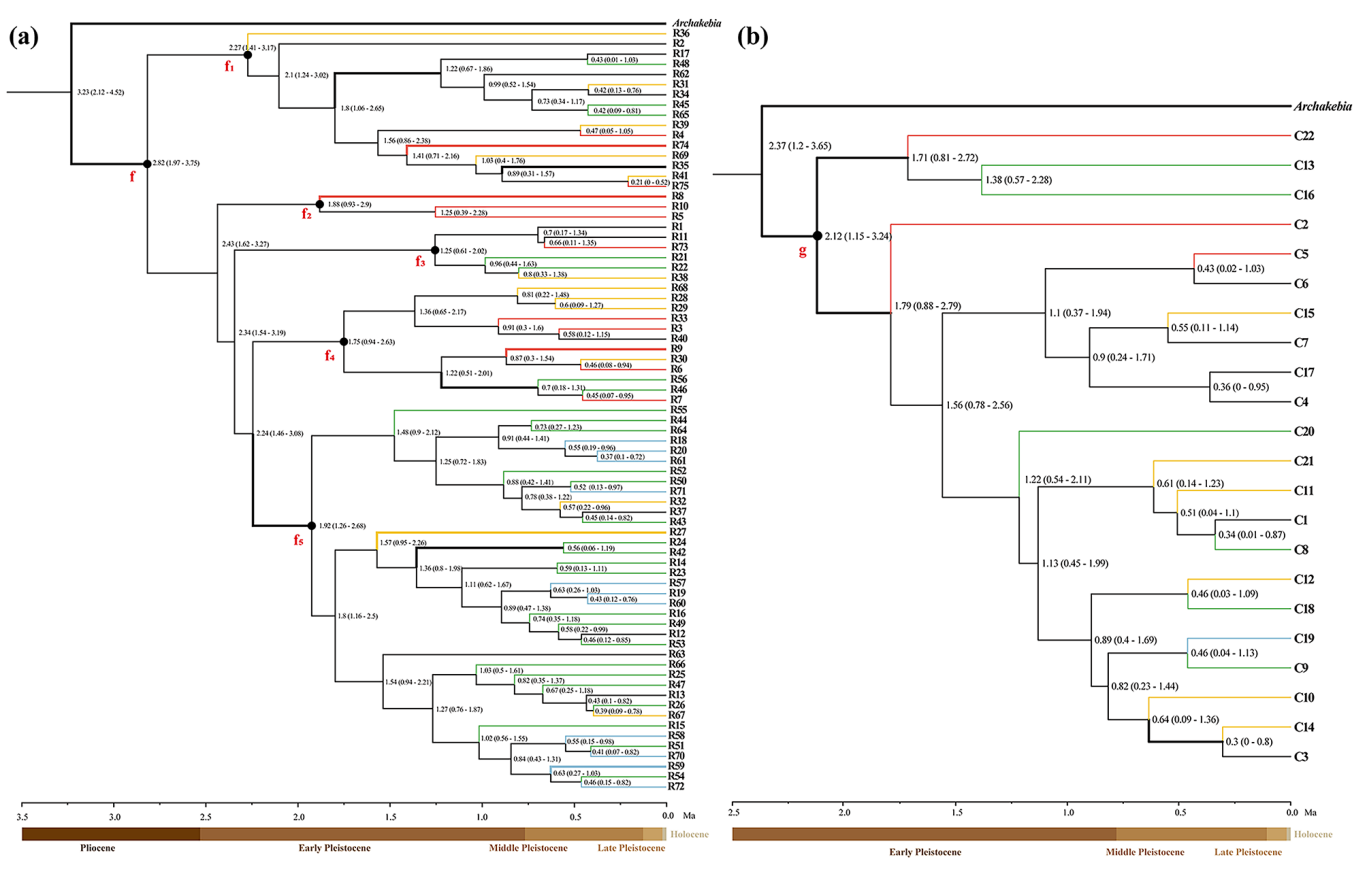
BEAST2-derived chronograms of ITS haplotypes (**a**) and *rps16* haplotypes (**b**) calibrated by the crown time of *Akebia trifoliata*. The age estimate (Ma) (HPD) for each node is shown beside the node. The regions with a greater than 95% posterior probability are indicated by thick branches. The red, yellow, green and blue branches represent haplotypes from the A (the eastern Tibetan Plateau), B (central Northern China), C (central China) and D (eastern China) regions, respectively, and the other black branches except those of the outgroup represent haplotypes from two to four different regions

The BEAST2-derived *matK* chronogram showed that the crown group age of *A. trifoliata* was 2.06 Ma (Fig. 4), and when it was used as a root prior, the coalescence times of 75 haplotypes (node f in Fig. 5a) and 22 haplotypes (node g in Fig. 5b) were 2.82 Ma and 2.12 Ma, respectively.

In the ITS chronogram, four nodes (f_1_-f_4_) covered 37 haplotypes: 12 specific to the A region, 10 specific to the B region, seven specific to the C region and eight non-region specific; node f_5_ covered 38 haplotypes: three specific to the B region, 21 specific to the C region, 10 specific to the D region, and four that were non-region specific.

The coalescence time of the five nodes varied from 2.27 Ma to 1.25 Ma, which falls within the early Pleistocene (2.58–0.78 Ma) (Fig. 5a), during which the divergence of all 22 ITS haplotypes and five *rps16* haplotypes occurred. However, the remaining 53 (70.67%) ITS haplotypes and 17 (77.27%) *rps16* haplotypes originated during the middle Pleistocene (0.78 − 0.12 Ma).

### Demographic history

**Fig. 6:**
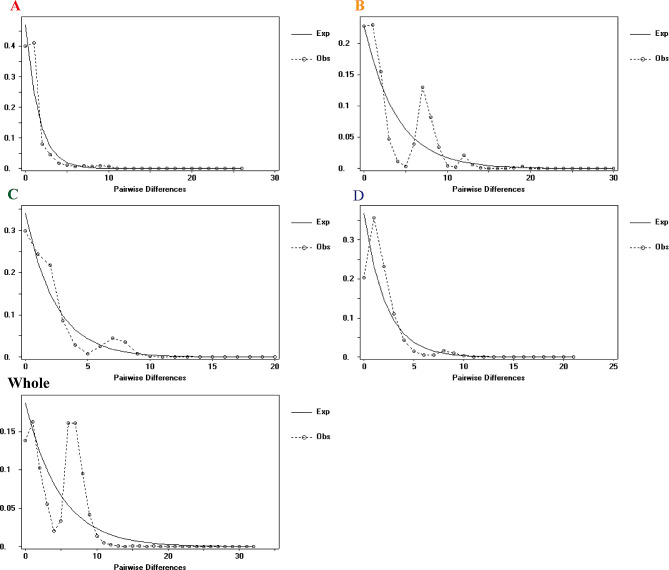
Mismatch distribution analyses of the five ITS clades consisting of both four regional populations and the whole population, in which the observed mismatch frequencies and best-fit curves of the sudden expansion model are shown

All neutrality test values, including Tajima’s D, Fu and Li’s D and Fu and Li’s F, of all ITS and *rps16* datasets in the whole population and all four regions were negative, although those of the *rps16* haplotypes were not significant in the D region (Table 2). The mismatch distribution of the ITS haplotypes was unimodal in the A and D regions (Fig. 6), while that of the *rps16* dataset was peak free in the whole population and each region (Fig. S1). The results of the fit test using the sudden expansion model showed that the SSD (0.0267) and HRag (0.322) values of the ITS haplotypes in the whole population were not significant at the *p* = 0.05 level, which suggested the occurrence of one expansion event (possibly 150 Ka). However, the *rps16* haplotypes of *A. trifoliata* mainly experienced balancing selection throughout their evolutionary history because there were no detectable SSD or HRag values in the whole population or any of the four geographical regions.

**Table 2:** The results of neutrality tests for various populations

Region	*rps16*			ITS		
	Tajima’s D	Fu and Li’s D	Fu and Li’s F	Tajima’s D	Fu and Li’s D	Fu and Li’s F
Whole	-2.68*	-10.36*	-8.08*	-2.00*	-7.04*	-5.479*
A	-2.58*	-7.289*	-6.53*	-2.52*	-7.33*	-6.44*
B	-2.61*	-5.27*	-5.13*	-1.87*	-2.44*	-2.66*
C	-1.94*	-3.63*	-3.59*	-2.01*	-4.01*	-3.81*
D	-1.27	-0.44	-0.44	-2.091*	-3.75*	-3.760*

*, *p* < 0.05

### Ancestral area reconstructions

**Fig. 7:**
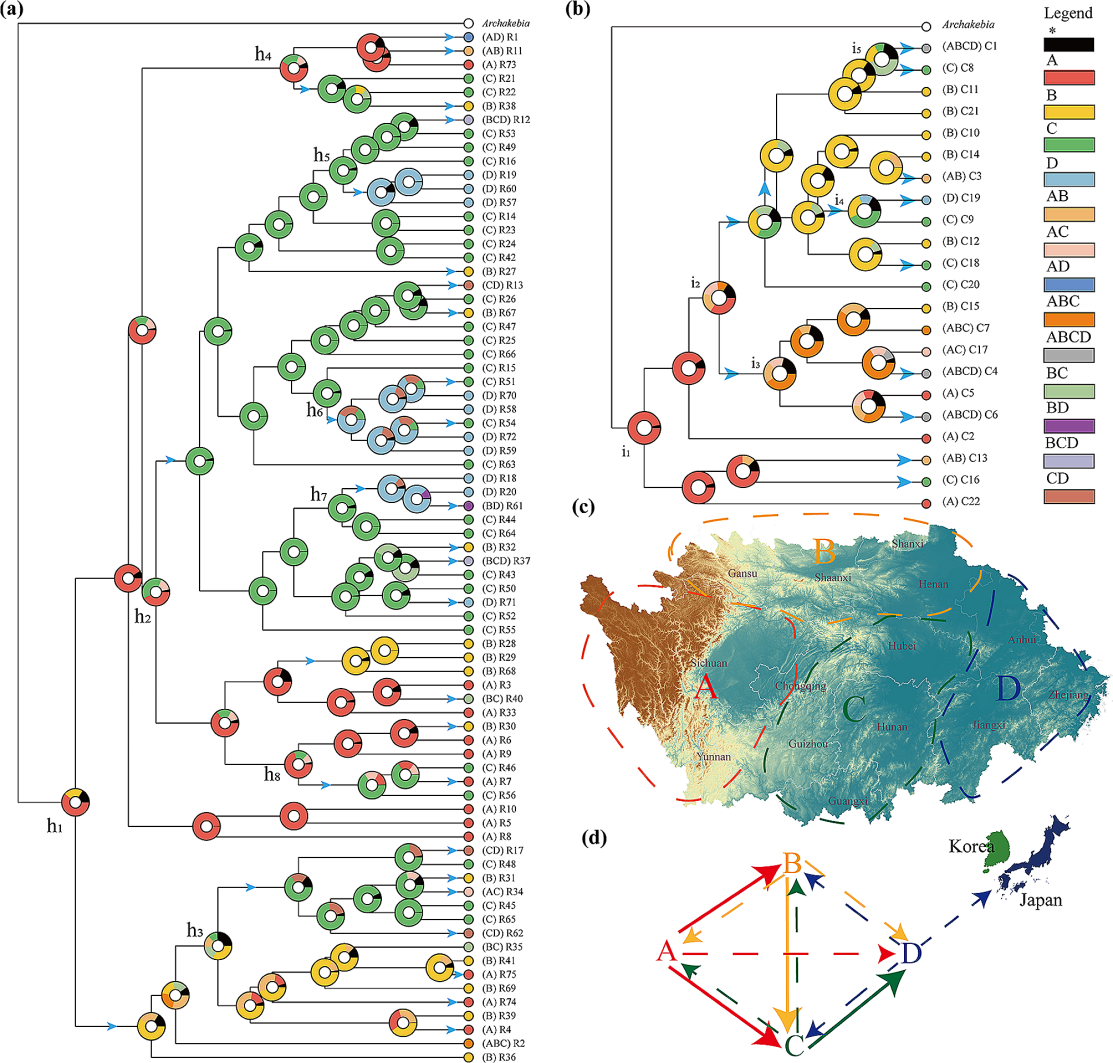
Ancestral area reconstructions based on the Bayesian binary Markov chain Monte Carlo (BBM) method performed in the RASP using the BEAST2-derived chronogram of *Akebia trifoliata* based on (**a**) ITS and (**b**)*rps16* haplotypes (see Fig. 5a and **b**). The circle at each node represents the marginal probability of each alternative ancestral region derived from the BBM analysis. The results are based on a maximum area of four. Inferred dispersal events are indicated by blue arrows. (**c**) Distribution area map of *Akebia trifoliata*: A (the eastern Tibetan Plateau), B (central northern China), C (central China) and D (eastern China). (**d**) Speculative diagram of expansion or propagation events in *Akebia trifoliata*, with bold solid lines representing the main expansion or propagation events

According to the topology of the reconstructed ancestral area of the ITS region, the marginal probabilities of the A region at h_1_, h_2_, h_4_ and h_8_ were 0.64, 0.45, 0.52 and 0.77, respectively (Fig. 7a). The populations of other regions, especially the C region, colonized from the A region. The marginal probability of B at h_3_ was the largest (0.25), which suggested that region C was also partly colonized by the population from region B. The marginal probabilities of C at h_5_, h_6_ and h_7_ were 0.97, 0.97 and 0.95, respectively, indicating that the population of the D region was largely derived from the C region.

Similarly, the marginal probability of the A region at i_1_ was 0.97 in the *rps16* topology (Fig. 7b), also suggesting that the A region was the ancient distribution area of *A. trifoliata*. The value (0.40) at i_2_ indicated that the populations of both the B and C regions could have colonized from the A region. The marginal probability of the C region at i_4_ was 0.39, suggesting that the population of the D region mainly colonized the C region. We also noticed that the information at both i_3_ and i_5_ was confusing, which could result from the unclear pedigree of *rps16*.

BBM analyses showed the potential for many seed dispersal events (see the blue arrow in Fig. 7), with only the absence of directional dispersal events from the population in the D region to the A region. Generally, the species migration route was from the ancestral distribution A region simultaneously to the B and C regions, then from the B region to the C region, and finally from the C region to the D region (Fig. 7c and d).

## Discussion

### The characteristics of the population and conserved DNA sequences help elucidate the phylogeographic structure of *A. trifoliata*

Genetic diversity parameters such as the h_T_, Hd and π of conserved DNA sequences provide the best evidence for elucidating the phylogeographic structure of a given species or a larger classification unit, such as genus, family and even order [40]; however, population characteristics, including geographical distribution, breeding system and effective size, also have an important influence on genetic diversity [60]. Whether we can accurately detect phylogeographic structure is largely dependent on two key factors: population characteristics and DNA sequence conservation.

Previous studies demonstrated that *A. trifoliata* originated in China and exhibited a constrained continuous distribution from the eastern edge of the TP to the eastern coast of mainland China and even to South Korea and Japan [28, 29]. In addition, the biological characteristics [30] and availability of sufficient germplasms via the ex situ living conservation method [31] have been reported. The 391 genotypes employed in this study originated from various places in mainland China, and there was a small geographic interval between adjacent samples (Fig. 2c), providing an ideal population for deducing the evolutionary history of species, especially climbing vine plants, in mainland China.

Molecular markers based on conserved DNA elements, including maternal chloroplast and biparental nuclear genomic markers, have been widely employed to investigate population genetic diversity. In this study, a previously reported chloroplast gene (*rps16*) and a nuclear DNA marker (ITS) were used to determine the genetic diversity of the *A. trifoliata* population [35, 36]. We first checked 22 and 75 haplotypes from 59 to 88 polymorphic sites in the *rps16* and ITS sequences, respectively (Table S1). Based on the haplotypes, the genetic diversity (h_T_=0.881, π × 10^− 3^=3.07) of *A. trifoliata* at the nuclear DNA level (Table S1) was greater than that of *Zingiber officinale* [61], *Pinus attenuata* [62] and *Antirrhinum charidemi* [63]. We found that the genetic diversity (h_T_=0.313, π × 10^− 3^=0.90) of the *A. trifoliata* population at the chloroplast sequence level was lower than that at the nuclear DNA level (Table S1), which could result from the low variation rate of the chloroplast compared with the nuclear DNA sequence [64]. This also provides a reasonable explanation for why the ITS and *rps16* sequences are used to determine the genetic structure of intraspecific and interspecific populations, respectively [63, 65]. To obtain more information on different DNA sequences, we simultaneously used *rps16* and ITS markers to determine genetic diversity in the present study, which would be beneficial for elucidating phylogeographic structure.

### *A. trifoliata* populations exhibited clear phylogeographic structure

Usually, molecular variation [66], the relationship between GGD and GD [67], the number of regional private haplotypes [19] and the relative sizes of G_ST_ and N_ST_ [20] are important information for describing population structure. First, we found that there was large molecular variation in the ITS sequence among different regional populations, while the variation in the *rps16* sequence was small (Table 1). Second, we also found a significant positive relationship between GGD and GD based on the ITS sequence, while there was no obvious relationship between them based on the *rps16* sequence (Fig. 1); in this study, the weak relationship could partly result from uneven samples from various regions (Table S1). Third, some regional private haplotypes of both ITS and *rps16* existed in each region (Fig. 2a and b; Table S1) and even on each branch of the ML tree (Fig. 3), possibly related to the high differentiation of different regions (Table 1). Finally, for the ITS sequence, the G_ST_ (0.437) was obviously lower than the N_ST_ (0.677). Taken together, these findings suggest that there was no obvious geographical structure at the chloroplast DNA level, possibly resulting from different inheritance modes [64], dispersal strategies [68] and slow variation rates because of their high conservation [65]; however, the clear phylogeographic structure of the *A. trifoliata* population at the nuclear DNA level is beyond all reasonable doubt.

### Multiple historical ice shelters could have affected the current distribution status of *A. trifoliata*

Studies have shown that plant populations around glacial habitats usually have high genetic diversity and many private alleles or haplotypes [17, 69]. Here, we found that many haplotypes of both ITS and *rps16* were region-specific (Figs. 2a and b and 3), which indicated that there were multiple refuge regions. According to the number of haplotypes, Hd and π, three putative main refuge regions (the Qinba Mountains, Nanling Mountains and Yunnan-Guizhou Plateau) can be suggested.

First, the Qinba Mountains could be the main refugia region of *A. trifoliata* because the populations, including Bazhong from the A region and both Ankang and Xian from the B region, had many ITS haplotypes, high Hd and large π, and they commonly showed the ancestral haplotype R2 (Fig. 2a), which was also well supported by the high polymorphism of *A. trifoliata* resources previously reported in the region [34]. Second, the Nanling Mountains, as representative mountains in the subtropical region and one of the major biodiversity hotspots in China [70], could also be a refuge region because the Hezhou, Yongzhou, Guilin, and Ganzhou populations from the C or D region had similarly many haplotypes and high Hd and π values (Fig. 2b), and R12 was their common haplotype. Various studies have suggested that the Nanling Mountains provide shelter for many plants [71], such as *Castanopsis eyrie* [72] and *Loropetalum chinense* [73], which supports the Nanling Mountains as a historically important refuge region for *A. trifoliata*. Third, the Yunnan-Guizhou Plateau is still a refuge region because of the very high π values for the *rps16* sequence in addition to the ITS sequence in both the Bijie and Zunyi populations from the C region [74, 75].

At present, the CE and ISS models represent two prevalent views about the contraction and expansion of plant species in the subtropical region of China during the ice age cycle [14, 20]. Unfortunately, the CE model largely ignores the existence of many similar ecological niches, while the ISS model overlooks the effects of migration on species distributions. In this study, among the 61 populations, 55 had a sample size larger than two, of which 26 populations had an Hd value of ITS greater than 0.6 (Table S1). Of these 26 populations, 12 were geographically close to the three main refuge regions, while 14 were far from the main refuge regions, which clearly showed that the three main refuge regions (the Qinba Mountains, Nanling Mountains and Yunnan-Guizhou Plateau) harboured many independent, small ice shelters. In addition, many haplotypes, including 53 ITS haplotypes and 17 *rps16* haplotypes, originated during the middle Pleistocene (0.78 − 0.12 Ma), while the other haplotypes were produced before this stage, which indicated that whole or partial population expansion occurred in the past. The results of the neutrality test and ITS mismatch distribution analysis clearly demonstrated that population expansion events occurred in at least the A and D regions. The view of three main refuge regions with some independent, small ice shelters provides a reasonable explanation for the continuous and wide distribution of *A. trifoliata* in many regions of mainland China.

### Putative migration route and differentiation events of *A. trifoliata*

Molecular dating can provide information about the differentiation time of conserved DNA sequences, which is helpful for inferring population differentiation processes [76]. In this study, molecular dating revealed that the differentiation times of the common ITS and *rps16* haplotypes in *A. trifoliata* were 2.82 Ma and 2.12 Ma, respectively (Fig. 5a and b), and both occurred during the period spanning the late Pliocene to early Pleistocene. During this period, the third uplift of the Qinghai‒Tibet Plateau [77], the Asian monsoon [78] and the Asian drought [79] gradually intensified, causing habitat fragmentation and species differentiation [80]. For example, the Hainan Island population of *Tetrastigma hemsleyanum* differentiated at 2.78 Ma [22]. Second, most (72.2%) of the haplotypes of *A. trifoliata* differentiated in the middle Pleistocene (0.78 − 0.12 Ma), which is similar to the haplotype differentiation time of *Sargentodoxa cuneata* in this region [76], but the remaining 27.8% of the *A. trifoliata* haplotypes differentiated before this period (Fig. 5a and b). Hence, the putative divergence times of the common ITS and *rps16* haplotypes in *A. trifoliata* could be reasonable. In addition, both the frequency and distribution of haplotypes (Fig. 2a and b) suggested that region A was the ancestral region because of the high frequency and wide distribution of the ancestral haplotypes R2 and C1 (Table S1).

The results of further BBM analysis revealed the putative migration route (Fig. 7c and d), and in this model, the route from the ancestral A region simultaneously from both the B and C regions, from the B region to the C region, and then from the C region to the D region was the main route of *A. trifoliata* migration, and the corresponding reverse route could be a minor migration route. In fact, this hypothesis of species migration off the Qinghai‒Tibet Plateau [81] also agreed well with the putative migration model. In addition, we also found minor migration from the ancestral A region to the D region, while there was no reverse route. Finally, migration from west to east could be a major direction, while reciprocal migration between the south and the north could be minor, which could have resulted from climate oscillation in the past [17]. The main route and direction of *A. trifoliata* migration could be closely related to its natural spread through west-east-flowing rivers, especially the Yangtze River [31], animals such as birds [82] and even commercial action [83]. Moreover, incomplete coverage of glaciers during this process [84] may also explain the widespread differentiation of *A. trifoliata*.

The last concern was the origin of the *A. trifoliata* distributed in Korea and Japan, and in our opinion, these *A. trifoliata* possibly migrated from the D region of mainland China, which could be resolved by a future study with samples from these two countries.

## Conclusions

Both conserved DNA sequences and continuously and widely distributed large populations provide a valuable opportunity to elucidate the evolutionary history of a given species. In this study, a maternally inherited chloroplast gene (*rps16*) and a biparentally inherited nuclear ITS sequence were simultaneously employed to assess the phylogeographic structure of *A. trifoliata* populations in the region across the QTP eastern edge and subtropical China, where more than 90% of publications about the species originated. Our results suggested that *A. trifoliata* originated on the eastern edge of the QTP and then migrated eastwards to the east coast of mainland China, even to Korea and Japan, and that multiple ice shelters in the Qinba Mountains, Nanling Mountains and Yunnan-Guizhou Plateau region supported its proliferation throughout subtropical China. Overall, the unique origin, long-distance migration, high intraspecific divergence, multiple ice shelters and regional expansion of *A. trifoliata* resulted in its continuous and wide distribution.

### Electronic supplementary material

Below is the link to the electronic supplementary material.

**Supplementary Table S1:** Locations of populations of *Akebia trifoliata* sampled, sample sizes(n), the cpDNA haplotype and ITS haplotype frequency, haplotype distribution type, haplotype (gene) diversity (Hd) and nucleotide diversity (π × 10 − 3) of each population

**Supplementary Table S2:** The taxa of Ranunculales used for estimating the crown group age of *Akebia trifoliata*

**Supplementary Figure 1:** Mismatch distribution analyses of the five *rps16* clades consisting of both four regional populations and the whole population, in which the observed mismatch frequencies and best-fit curves of the sudden expansion model are shown

## Data Availability

All the original DNA data, including the *rps16* and ITS sequences of *A. trifoliata* used in this study, were uploaded to the GenBase of the Chinese National Genomics Data Center (NGDC) under the accession numbers C_AA025186.1 ∼ C_AA025576.1 for the ITS dataset and C_AA024795.1 ∼ C_AA025185.1 for the *rps16* dataset.

## Abbreviations

*A. trifoliata*: *Akebia trifoliata*
BBM: Bayesian binary MCMC
CE model: Contraction and postglacial expansion model
CTAB: Cetyltrimethylammonium bromide
DNA: Deoxyribonucleic Acid
ISS model: In situ survival model
ITS: Internal transcribed spacer
LGM: Last glacial maximum
ML: Maximum likelihood
PCR: Polymerase chain reaction
QTP: Qinghai‒Tibet Plateau
*rps16*: Ribosomal protein S16
SSD: Sum of squared deviations
TP: Tibetan Plateau
