# Supplementary material for: Conserved DNA sequence analysis reveals the phylogeography and evolutionary events of Akebia trifoliata in the region across the eastern edge of the Tibetan Plateau and subtropical China
Source: BMC Ecol Evol. 2024 Apr 23;24:52. doi: 10.1186/s12862-024-02243-0 (PMC11040917; doi:10.1186/s12862-024-02243-0)

Fig. S1. Mismatch distribution analyses of the five *rps16* clades consisting of both four regional populations and the whole population, in which the observed mismatch frequencies and best-fit curves of the sudden expansion model are shown.

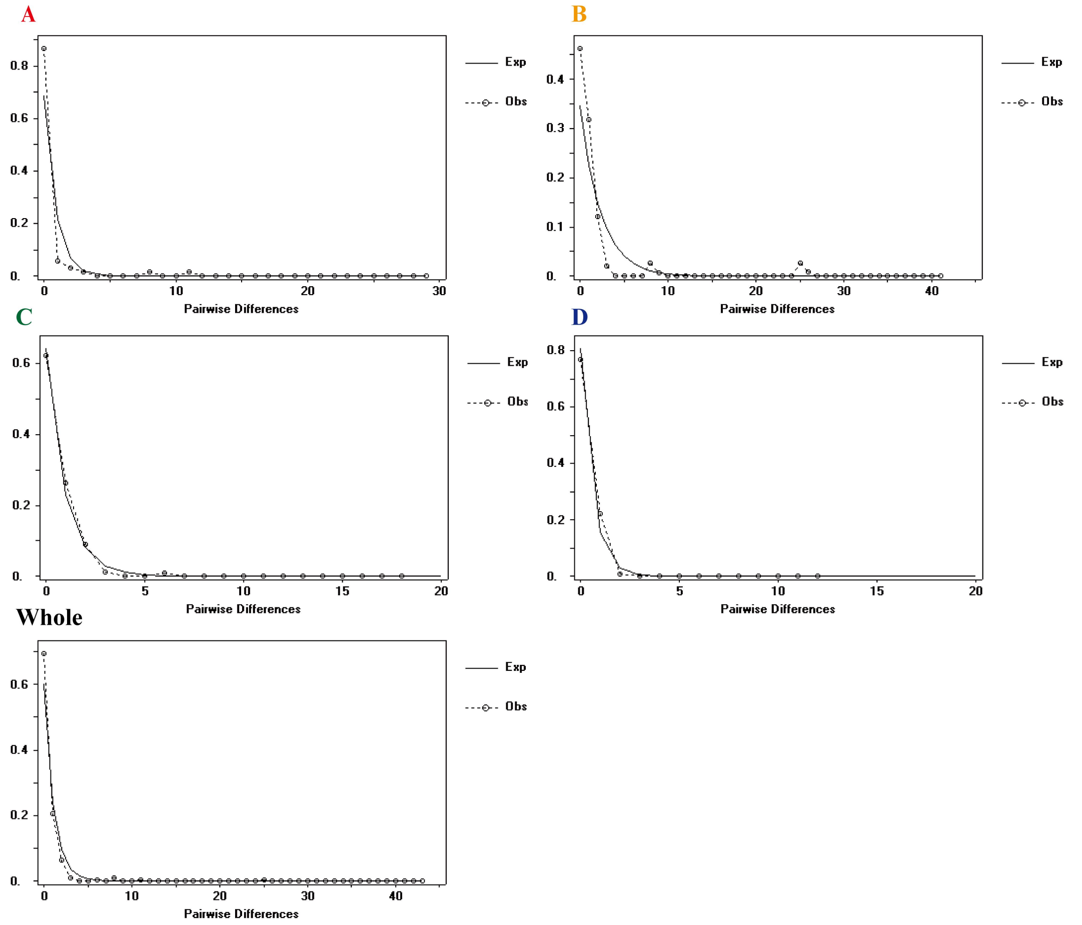

Supplement: Supplementary file 3 — Supplementary Figure 1: Mismatch distribution analyses of the five rps16 clades consisting of both four regional populations and the whole population, in which the observed mismatch frequencies and best-fit curves of the sudden expansion model are shown [file 12862_2024_2243_MOESM3_ESM.pdf]
